# Neonatal Sepsis and Inflammatory Mediators

**DOI:** 10.1155/2014/269681

**Published:** 2014-12-30

**Authors:** Juliana Reis Machado, Danilo Figueiredo Soave, Marcos Vinícius da Silva, Liliana Borges de Menezes, Renata Margarida Etchebehere, Maria Luiza Gonçalves dos Reis Monteiro, Marlene Antônia dos Reis, Rosana Rosa Miranda Corrêa, Mara Rúbia Nunes Celes

**Affiliations:** ^1^Department of Pathology, Institute of Tropical Pathology and Public Health, Federal University of Goias, 74605-050 Goiania, GO, Brazil; ^2^Department of General Pathology, Institute of Biological and Natural Sciences, Federal University of Triangulo Mineiro, 74605-050 Uberaba, MG, Brazil; ^3^Department of Pathology, Faculty of Medicine of Ribeirao Preto, University of Sao Paulo, 14049-900 Ribeirao Preto, SP, Brazil; ^4^Laboratory of Immunology, Institute of Biological and Natural Sciences, Federal University of Triangulo Mineiro, 38025-180 Uberaba, MG, Brazil; ^5^Surgical Pathology Service, Clinical Hospital, Federal University of Triangulo Mineiro, 38025-180 Uberaba, MG, Brazil

## Abstract

Neonatal sepsis is a major cause of morbidity and mortality and its signs and symptoms are nonspecific, which makes the diagnosis difficult. The routinely used laboratory tests are not effective methods of analysis, as they are extremely nonspecific and often cause inappropriate use of antibiotics. Sepsis is the result of an infection associated with a systemic inflammatory response with production and release of a wide range of inflammatory mediators. Cytokines are potent inflammatory mediators and their serum levels are increased during infections, so changes from other inflammatory effector molecules may occur. Although proinflammatory and anti-inflammatory cytokines have been identified as probable markers of neonatal infection, in order to characterize the inflammatory response during sepsis, it is necessary to analyze a panel of cytokines and not only the measurement of individual cytokines. Measurements of inflammatory mediators bring new options for diagnosing and following up neonatal sepsis, thus enabling early treatment and, as a result, increased neonatal survival. By taking into account the magnitude of neonatal sepsis, the aim of this review is to address the role of cytokines in the pathogenesis of neonatal sepsis and its value as a diagnostic criterion.

## 1. Introduction

Sepsis is one of the most common infectious conditions during the neonatal period, and it is still a significant cause of morbidity and mortality, despite the outstanding development of neonatology in recent years [1]. Described as systemic inflammatory response (SIRS) associated with a suspected or proven infection, the sepsis is an infectious disease of varied etiology, which determines degrees of inflammatory and metabolic responses [2, 3]. Tumor necrosis factor (TNF), interleukin-1 (IL-1), interleukin-6 (IL-6), and interleukin-8 (IL-8) are proinflammatory cytokines, whereas interleukin-10 (IL-10) and transforming growth factor-beta (TGF-*β*) are known as anti-inflammatory cytokines, both produced rapidly in the setting of neonatal sepsis. In the past years, several authors have supported the use of cytokines in the diagnosis of both early and late sepsis [4].

Previous results from our study, in which cytokines were measured in the plasma and in the umbilical cord blood at birth, support the idea that increased levels of either proinflammatory cytokines (TNF-*α*) or anti-inflammatory cytokines (IL-10) in neonates at birth change throughout the infectious process and there is also a positive and significant correlation between the levels of these cytokines [5]. In the present review, we will cover the actual role of cytokines in the pathogenesis of neonatal sepsis and its value as a diagnostic criterion.

## 2. Neonatal Sepsis

Neonatal sepsis is a systemic infection that occurs in newborns up to 28 days of age and it is a major cause of morbidity and mortality in newborns [6]. According to World Health Organization, in 2010, 3.7 million newborns died before reaching 28 days of age in the United States, and 37% were due to infectious causes [7]. As proposed by The International Pediatric Sepsis Consensus Conference in 2002, specific definitions for pediatric SIRS and sepsis present important differences related to clinical signs and laboratory biomarkers specific to adults. The major differences between adults and children are that the diagnosis of pediatric SIRS requires lower values for heart rate, leukocyte count, and systolic blood pressure and upper values for heart rate, respiration rate, or leukocyte count. In Sepsis, to confirm the diagnosis, it is necessary the presence of bacterial infection suspected or confirmed by culture or other methods. Taken together, some clinical findings can also help the diagnosis process such as: petechiae and purpura (in the setting of hemodynamic instability); fever, cough, and hypoxemia (in the setting of leukocytosis and pulmonary infiltrates) [3].

In adults, sepsis is defined as a complex clinical syndrome of severe systemic inflammatory response syndrome (SIRS) with multiple physiological and immunological abnormalities, which is usually associated with bacterial or fungal infections [2, 8–10]. Sepsis pathogenesis is associated with hemodynamic changes, disturbances of microcirculation and cellular changes that cause imbalance between blood flow and metabolic tissue requirements, leading to multiple-organ dysfunctions, which is responsible for the severe and often fatal form of the disease [11]. Neonatal sepsis and sepsis in adults are very different conditions, with implications for the epidemiology and pathophysiology and even in clinical management. In addition, differences between neonates and adults directly impact the involvement of cytokines during development of sepsis and the use of assessment of these mediators in clinical routine [12–14]. Although our focus here is neonatal sepsis, as far as possible we compare results of studies involving adults from those obtained from studies involving neonates.

Sepsis development can be initiated through recognition of one or more components of invading organism, including structural elements such as Gram-negative endotoxins or secreted exotoxins that stimulate the local and systemic release of endogenous inflammatory mediators. Among the inflammatory mediators are cytokines such as TNF-*α*, IFN-*γ*, IL-1*β*, IL-6, and IL-8, which act favoring the migration and activation of immune cells [15, 16]. Stimulation of polymorphonuclear leukocytes, histiocytes, platelets, and endothelial cells leads to the production of biologically active mediators, including platelet activating factor (PAF), arachidonic acid metabolites, histamine, bradykinin, complement proteins, vasoactive peptides, and oxide nitric (NO). The production and release of these proinflammatory mediators can induce a systemic inflammatory response characteristic of the initial phase of sepsis [17, 18].

For a long time, it was believed that sepsis was caused by an exacerbated inflammatory response generated from innate immune response triggered by bacterial infections. However, researchers eventually described the presence and importance of “compensatory anti-inflammatory response syndrome,” CARS [13], which often occurs after hyperinflammatory phase, especially in patients who develop severe sepsis [19].

In neonates, severe sepsis is characterized by the persistence and prevalence of proinflammatory mediators up to the third day after diagnosis, whereas these groups of cytokines are prevalent in sepsis, with good clinical evolution, just on the day of diagnosis [4]. The study of inflammatory mediators and cytokines as biomarkers of neonatal sepsis are really important for syndrome diagnosis. Recent research has pointed to the role of damage-associated molecular patterns (DAMPs), which are intracellular proteins released in response to cell injury, such as high mobility group box protein 1 (HMGB-1) [20]. DAMPs act as endogenous danger signals to activate and amplify the function of receptors such as receptor for advanced glycation end products (RAGE), hence perpetuating the inflammatory response [21]. This may be particularly important for neonatal sepsis, because the maternal-fetal barrier is compromised by the inflammatory response, leading to translocation of DAMPs into the fetus [22].

The study of proinflammatory cytokines IL-6, TNF-*α*, IL-1*β*, and IL-8 and anti-inflammatory cytokines IL-10 and TGF-*β* as reliable biomarkers of neonatal infection has been shown to be potentially useful for early sepsis diagnosis and to predict the severity of disease at early stages of the infection [23–26].

## 3. Cytokines

Cytokines are relatively small molecules with short serum half-life (from minutes to a few hours) and play a central role in immune response in neonates with sepsis. During sepsis, cytokine levels may be observed in picograms per milliliter of plasma or in nanograms or even micrograms per milliliter [27].

In the 1990s, sepsis was believed to be associated with an exacerbated release of mainly proinflammatory cytokines, such as tumor necrosis factor (TNF-*α*), interleukin (IL-1, IL-6, and IL-12), interferon-*γ* (IFN-*γ*), and macrophage migration inhibitory factor (MIF). The expression “cytokine storm” thus arose [28]. However, recent research on the pathophysiologic mechanisms underlying sepsis indicates that the profound proinflammatory response is counteracted by certain anti-inflammatory cytokines, including IL-10, transforming growth factor (TGF-*β*), and IL-4, which attempt to restore immunological balance [18, 29, 30].

### 3.1. Interleukin-6 (IL-6)

Interleukin-6 (IL-6) is the most studied cytokine in neonatal population. IL-6 is a cytokine that shows early response to infection, preceding the increase in C-reactive protein and followed by TNF-*α* release. It is synthesized by mononuclear phagocytes, endothelial cells, fibroblasts, and the decidua, chorion, amnion, and trophoblast cells soon after stimulation by microbial products [31, 32].

IL-6 acts as a signal in the activation of T cells, and it induces the secretion of antibodies by B cells and the differentiation of cytotoxic T cells. It stimulates the release of other cytokines, particularly TNF-*α* and IL-1*β*. IL-6 is an early marker in the diagnosis of neonatal sepsis, increasing several hours before the increase in C-reactive protein. The sensitivity of these tests together can reach values close to 100%, hence the importance of these markers [33].

Interleukin-6 is considered the major inducer of the hepatic protein synthesis. It is capable of interfering with the production of C-reactive protein, so it can be detected earlier than C-reactive protein during bacterial infection. The C-reactive protein has both anti-inflammatory and proinflammatory effects during infection, since it mediates the elimination of pathogens, despite also inhibiting the interaction between endothelial cells and leukocytes. As the secretion starts 4–6 hours after stimulation and it peaks at 36 hours after infection, it is often used in the diagnosis of infection [34].

IL-6 is not considered a “gold standard” biomarker to have a very short half-life, approximately 100 minutes in patients with meningococcal infection [35]; additionally, the circulating levels decrease or return to basal levels 24 hours after appropriate treatment in late-onset neonatal sepsis [36]. Several studies have reported sensitivity for detection of IL-6 in 75 to 90% of circulating serum in the first 24 hours of infection, with a marked reduction in the diagnostic effectiveness 48 hours after the onset of symptoms and suspected sepsis [23, 25, 37, 38]. Moreover, IL-6 has been correlated with maternal chorioamnionitis and used in the initial diagnosis of early neonatal sepsis when detected at high levels in umbilical cord blood [39]. Similar results were found by our research group when analyzing liver of perinatal deaths diagnosed with fetal inflammatory response syndrome (FIRS) using immunoperoxidase method; we observed that IL-6 and C-reactive protein were overexpressed [40].

When detected in the umbilical cord blood of newborns at term and without risk factors, IL-6 does not have significant clinical utility in differentiating infected and healthy newborns [41]. On the other hand, in newborns with premature rupture of membranes, IL-6 showed high sensitivity and specificity in predicting funisitis and positive cord blood culture [31].

The difference in IL-6 levels in cord blood and blood of the newborn infant at birth is due to the kinetics of IL-6. Therefore, sample collection time is an important factor for the detection of high levels of IL-6 in neonates [42].

### 3.2. Tumor Necrosis Factor Alpha (TNF-*α*)

Macrophages and monocytes appear to be the major cellular source of TNF-*α* [35]. Release of TNF-*α* may occur approximately 30 minutes after LPS injection, and the circulating levels reach their peak in approximately one and a half hour [43], with an estimated half-life of about 70 minutes [30, 32]. Tumor necrosis factor (TNF-*α*) is the prime mediator of septic shock in neonates and widespread tissue injury, and it regulates the secretion of IL-1*β* [37, 38]. High levels of TNF-*α* appear to be related to the severity of the disease, although some studies in adults do not confirm this relationship [44, 45]. The peak plasma concentration of TNF-*α* is reached after an hour of experimental endotoxemia, with near-zero levels for three hours [46]. There are evidences that TNF-*α* is found free in plasma concomitant with the appearance of signs and symptoms of bacterial infection [16].

Systemic release of TNF-*α* can cause vasodilation and increased vascular permeability leading to systemic edema, with decreased blood volume and hypoproteinemia that can progress to shock. There was stimulus for leukocyte and platelet adhesion, with clots formation in small vessels and consumption of coagulation proteins that may lead to disseminated intravascular coagulation. This condition may also progress to multiple-organ failure and death in early-onset neonatal [47, 48].

In addition, TNF-*α* and IL-1 were identified as crucial cytokines to development of fever and, therefore, belong to a group of pyrogenic cytokines [30, 49]. TNF-*α* induces increased adherence of neutrophils and integrins to endothelial tissues and upregulates the endothelial expression of procoagulant proteins. Moreover, together with IL-1, TNF-*α* was one of the first mediators identified in inflammatory sites [50]. Synergically, TNF-*α* and IL-1 amplify the inflammatory signals by activating macrophages to produce proinflammatory cytokines such as IL-6 and IL-8, as well as lipid mediators and reactive oxygen species [18] leading to sepsis-induced organ dysfunction. TNF-*α* acts through two receptors, TNFR1 (TNF receptor-1) and TNFR2 (TNF receptor-2), resulting in immune cell activation and release of several immunoregulatory mediators [30].

The role of TNF-*α* as a marker for the prediction of early-onset neonatal sepsis has been suggested. Additionally, when used in combination with IL-6, TNF-*α* may achieve up to 98.5% sensitivity [38]. On the other hand, Santana and colleagues [51] demonstrated that TNF-*α* was not significantly different between sick and healthy neonates. Discrepancy between the studies could be due to the fact that the kinetics of TNF-*α* production is not fully understood in early life.

Furthermore, due to the short half-life of TNF-*α* and its interaction with soluble receptor, the detection becomes difficult [52]. Thus, TNF-*α* as a reliable biomarker for sepsis is compromised. In contrast, some studies, especially by Pickler and colleagues [53], reported that high levels of TNF-*α*, IL-6, and IL-1 are often associated with profiles of sepsis in neonates. These authors also confirmed the low sensitivity and specificity of TNF-*α* to differentiate sepsis in infected newborns.

Experimental studies have shown that the injection of TNF causes a syndrome which is indistinguishable from septic shock [54]. It was observed that the infusion of recombinant TNF-*α* in humans results in SIRS [55–57]. It is believed that because this role in the inflammatory process results in severe clinical conditions of sepsis, TNF-*α* may have a direct correlation with the severity of sepsis and the mortality rate during the development of sepsis in newborns at risk for infections [58].

### 3.3. Interleukin-1*β* (IL-1*β*)

IL-1 is a proinflammatory cytokine released by timely activated macrophages similar to those of TNF-*α*. It signals through two distinct receptors, called IL-1 receptor type I (IL-1R1) and IL-1R2, and has many effects on immune cells [30, 59].

In the inflammatory response cascade, IL-1*β* and TNF-*α* induce IL-6 release by the endothelial cells. Other cytokines, such as TNF-*α*, can also mediate the production of IL-1*β* [60]. Namely, IL-1*β* can be produced by the central nervous system, particularly in the hypothalamus, and also can be induced by infectious agents (bacterial endotoxins, viruses, fungi, and parasite antigens) and C5a complement, usually in one hour, reaching peak levels in 5–10 hours [61, 62].

During sepsis, IL-1 seems to induce fever, coagulation, and hematopoiesis, promoting the extravasation of inflammatory cells [30]. Therefore, it has been noticed that IL-1 is significantly increased in most patients with sepsis, and it has been associated with the severity of sepsis [63, 64]. Moreover, persistently elevated levels of this cytokine have been correlated with the development of multiple-organ failure and with a worse prognosis in adults [45, 64].

Interleukin-1 has been described as a marker of neonatal sepsis, although its diagnostic efficacy is lower than that of IL-6 and TNF-*α* [24]. The diagnostic value of IL-6, TNF-*α*, and IL-1*β* is limited by the time of blood sample collection, which should be as early as possible if neonatal sepsis is suspected, since these cytokines have very short half-life [46, 65].

Therefore, analysis of a panel of cytokines, and not only the isolated measurement of a single cytokine, is required in order to characterize the inflammatory response in sepsis.

### 3.4. Interleukin-8 (IL-8)

IL-8 belongs to the class of proinflammatory chemokines and it is produced by the placental cells, fetal monocytes/macrophages, and cells. It is often produced after an infectious process originated in the uterus [32]. IL-8 is a chemokine which follows a course of time similar to that of IL-6. This characteristic limits, to a great extent, the role of IL-6 and IL-8 as clinically useful biomarkers for all stages of sepsis, although they may be useful early in the disease prior to treatment in neonates [66].

Recently, the association of high levels of IL-8 in the presence of retinopathy was determined in seventy-four very low birth weight preterm infants with clinical criteria of early infection whose cytokines were obtained during the first three days of life. The cut-off points for IL-6 >357 pg/mL, IL-8 >216 pg/mL, and TNF-*α* >245 pg/mL were significantly associated with the development of retinopathy [67].

Placental cells, fetal monocytes/macrophages, and endothelial cells are able to produce IL-8 after infectious process originated in the uterus. IL-8 levels increase about 90 minutes after infection and peak at about 120 minutes in septic neonates [68], whereas its circulating concentration decreases significantly 48 hours after birth, likewise the kinetics of IL-6 [69].

Gestational age has little effect on the umbilical cord blood IL-8 concentrations. Only preterm infants less than 32 weeks of gestation may have increased levels of IL-8 due to gestational age [70]. Data by Dembinski and colleagues [71] showed that IL-8 levels were undetectable in the umbilical cord blood of healthy newborns. In the past years, IL-8 has been extensively investigated as a predictive biomarker of early-onset neonatal sepsis [69] that is corroborated by Døllner and colleagues [41], who observed increased IL-8 levels in the umbilical cord blood of infected preterm neonates. The disadvantage of IL-8 measurement in comparison with IL-6 levels is the limit of detection. The IL-6 serum detection limit is >0.7 pg/mL, whereas the serum detection limit of IL-8 is >10 pg/mL [4].

### 3.5. Interleukin-10 (IL-10) and Transforming Growth Factor-Beta (TGF-*β*)

Anti-inflammatory cytokines, such as IL-10 and TGF-*β*, are important inflammatory mediators, since they play a major role in preventing excess proinflammatory response during sepsis [72]. IL-10 is produced by different types of immune system cells such as monocytes, macrophages, T and B lymphocytes, and NK cells [73]. This cytokine suppresses the production of proinflammatory mediators including TNF-*α*, IL-1, IL-6, IFN-*γ*, and GM-CSF in cells of the immune system [74].

IL-10 has been associated with septic shock in both adults and children [75]. High levels of IL-10 have been correlated with poor prognosis of sepsis in adults, shown to be a useful predictor of severity in septic shock and death [76, 77]. However, it was shown that the appropriate response of IL-10 may have a protective effect on SIRS and that high IL-6/IL-10 ratio was found in patients with a worse prognosis [78]. Similarly, a high IL-10/TNF ratio has also been associated with severe late-onset neonatal sepsis [26, 75].

In an experimental model, it was shown that the administration of recombinant murine IL-10 protects from lethal endotoxemia, even when IL-10 was injected 30 minutes after LPS administration [79]. In contrast, the immunoneutralization of IL-10 led to increased levels of TNF and IL-6 in circulating mice [80], and it also reversed the ability of IL-10 to protect mice from lethal endotoxin [79]. Despite these clear protective effects of IL-10 in LPS-induced diseases, IL-10 actions are not always beneficial [81]. The effects of IL-10 appear to depend on the time of administration in case of neutralizations [82]. Furthermore, the authors reported that IL-10-deficient mice showed an earlier onset of lethality after experimental sepsis induced by cecal ligation and puncture as compared with wild type mice [83].

TGF-*β*, as well as IL-10, is a member of the growth factor family and it is an important anti-inflammatory cytokine. TGF-*β* was shown to play a role in tissue repair and fibrosis [83], as well as in sepsis-induced immunosuppression [84]. In vitro, TGF-*β* suppresses the release of proinflammatory mediators such as IL-1*β* and TNF-*α* from monocytes and macrophages [85]. TGF-*β* also inhibits T-lymphocyte functions, such as IL-2, and the secretion of T cell proliferation [86], as well as promoting the development of T regulatory cells [87].

Studies involving evaluation of in vitro assays, experimental models of sepsis, and human clinical evaluations support the anti-inflammatory actions of TGF-*β*. These experiments have shown that treatment with TGF-*β* blocked endotoxin-induced hypotension, probably inhibiting the hypotensive effects of NO and improved survival in a rat model of Salmonella endotoxin-induced septic shock [72, 88].

Another study reported that adult patients at the onset of sepsis presented high levels of TGF-*β* even though these levels were not correlated with severity or prognosis of disease [89]. Recent data have demonstrated that TGF-*β* reverses the depression of cardiac myocyte contraction, which is induced by cytokines such as TNF-*α* and IL-1 and by the serum of patients with septic shock [90]. Hence, it is suggested that TGF-*β* may have cardioprotective effects in sepsis-induced cardiac injury.

## 4. New Perspectives of Biomarkers in Sepsis

Recently, IL-7, which is a hematopoietic growth factor, has been reported to have antiapoptotic roles, essential for lymphocyte survival and growth [91, 92]. IL-7 is produced by stromal cells in lymphoid tissues [93]. In addition to its antiapoptotic properties, it induces proliferation of CD4 and CD8 T cells. One of the characteristic features of sepsis is the profound loss of T cells in various lymphoid organs.

During experimental sepsis, IL-7 decreases cell apoptosis through the expression of antiapoptotic Bcl-2 gene [94, 95]. Another study showed that septic mice treated with recombinant IL-7 (rhIL-7) increase the local and systemic production of neutrophils and IL-17, thus recruiting more neutrophils to the site of infection [96]. Nevertheless, further studies are necessary to understand the real role of IL-7 in the pathogenesis of neonatal sepsis.

Another cytokine which has been studied and seems to be really involved in the pathogenesis of sepsis is IL-22 [95]. In a small group of hospitals within a single health center, IL-22 levels were slightly increased in the serum of patients with sepsis. It is believed that IL-22 that is produced during sepsis may contribute to host defense and to stabilizing mucosal barrier functions under systemic infection conditions [97]. However, the adverse effects of IL-22 are also described in a model of polymicrobial peritonitis, in which the levels of IL-22 and its receptor in the spleen and kidney were very high.

The biological activity of IL-22 is modulated by its antagonist, IL-22 pb. Treatment of mice with IL-22 pb before sepsis led to increased accumulation of neutrophils and mononuclear phagocytes, as well as a reduction in bacterial burden at the site of infection [98]. Thus, the beneficial effects of IL-22 are mediated by promoting tissue protection, whereas ill effects are connected with exacerbated inflammation. This dual role of IL-22 implies that it participates in the pathophysiology of experimental sepsis [95].

IL-33 is the newest member belonging to the IL-1 cytokine family. IL-33 can induce T helper cells, mast cells, eosinophils, and basophils to produce Th2 cytokines [99]. IL-33 mediates its biological effects through interaction with its receptors and with associated proteins abundantly expressed on the surface of Th2 cells and mast cells [99]. IL-33 also functions as a chemotactic mediator for Th2 cells [100, 101]. In mast cells, IL-33 triggers the production and release of proinflammatory cytokines, promotes maturation, and induces degranulation [102]. Furthermore, IL-33 amplifies the polarization of alternatively activated macrophages and it enhances TLR4-mediated cytokine production by macrophages [103]. ST2 exists in different splice variants, which results in a cell membrane-bound form and in a soluble form. The soluble form, sST2, is generated by alternative splicing and does not induce signaling, thus acting as a receptor for IL-33 [104]. High levels of sST2 have been associated with the pathogenesis of sepsis, so sST2 may be a potential marker of poor prognosis [95, 105]. IL-33 has beneficial effects in experimental sepsis, enhancing the accumulation of neutrophils through the upregulation of CXCR2 via GRK2-dependent pathways at the site of infection and reducing the inflammatory response in systemic sites. However, it has not yet been determined whether the administration of IL-33 actually represents a therapeutic strategy [95].

IL-17 family cytokines are important mediators of the immune response [22]. The proinflammatory cytokine IL-17A is mainly produced by Th17 cells and is involved in the mediation of proinflammatory responses, hence triggering the production of many other cytokines such as IL-1, IL-6, and TNF-*α* [106]. It has recently been shown that increased IL-17A levels have adverse effects during experimental sepsis in CLP-induced sepsis models. Whereas the blocking of IL-17A was associated with reduced levels of bacteremia, proinflammatory cytokines and an increased survival rates of animals [107].

IL-4 is the major cytokine produced by Th2 lymphocytes. It causes an increased production of IL-4 itself and of other anti-inflammatory cytokines, suppressing the secretion of monocyte-derived proinflammatory cytokines. Experimental studies have shown that IL-4 increases the survival rates of mice exposed to lethal LPS doses [108]. Similarly, in humans, mRNA expression of IL-4 was associated with survival of patients with severe sepsis. Nonetheless, IL-4 plasma levels of septic patients on the day of hospital admission were not different between the patients who survived and those who did not survive sepsis [109]. It was recently suggested that polymorphisms in the IL-4 gene promoters may affect the balance of Th1 and Th2 response and, consequently, predispose trauma patients to develop sepsis [110]. Although there are several studies suggesting that IL-4 plays an important role in the pathogenesis of sepsis, its real role in the course of the disease remains unknown.

Procalcitonin (PCT) is the hormone calcitonin, which is normally produced by the C cells of the thyroid gland, leading to massive release of PCT into the bloodstream depending on the severity of sepsis [111]. Assicot and colleagues [112] were the first to describe PCT as a potential biomarker of sepsis and infection. The authors showed a more favorable PCT kinetic profile than the profile of C-reactive protein and cytokines. PCT circulating levels decrease within about 24 hours, when the infection is sufficiently treated. Decreasing PCT levels are, thus, associated with improved survival, whereas increased or persistently increasing PCT levels are predictive of an unfavorable outcome [113, 114].

Discrimination between infectious and noninfectious conditions for PCT and decreasing PCT levels in properly treated patients raised the hypothesis that PCT levels can help in determining the antimicrobial therapy [34]. PCT serum concentrations may be increased in medullary thyroid carcinoma in the absence of bacterial infections and in conditions such as severe trauma, surgery, or postcardiac arrest, heat shock, stress birth, and various types of immunotherapies and some autoimmune diseases. Thus, PCT can guide the diagnosis of sepsis and management of antimicrobial therapy. However, as any other biomarker, PCT levels must be evaluated within the clinical context of the patient [34].

## 5. Conclusion

Understanding the neonatal sepsis pathogenesis still remains a challenge, given its complexity and the inherent immunological characteristics of the newborn. Cytokines seem to be one of the major mediators involved in the outcome of this entity. The imbalance between proinflammatory and anti-inflammatory cytokines appears to be related to both the severity and prognosis of neonatal sepsis. Therefore, the use of cytokines as biomarkers of neonatal sepsis seems plausible and necessary, since the early diagnosis of neonatal sepsis directly influences the therapy and prognosis.
